# Replicated Differential Expression Analysis in a Green‐Brown Polymorphic Grasshopper Reveals Role of Beta‐Carotene‐Binding Protein in Body Coloration

**DOI:** 10.1111/mec.70142

**Published:** 2025-10-22

**Authors:** Chongyi Jiang, Mahendra Varma, Abhijeet Shah, Octavio M. Palacios‐Gimenez, Holger Schielzeth

**Affiliations:** ^1^ Population Ecology Group, Institute of Biodiversity, Ecology and Evolution Friedrich Schiller University Jena Jena Germany; ^2^ Max Planck Institute for Chemical Ecology Jena Germany; ^3^ German Centre for Integrative Biodiversity Research (iDiv) Halle‐Jena‐Leipzig Leipzig Germany

**Keywords:** beta‐carotene‐binding protein (βCBP), body coloration, differential gene expression analysis, Gomphocerinae, green‐brown polymorphism, orthoptera

## Abstract

Orthoptera provide a well‐documented case of transspecies colour polymorphism, with green and brown morphs coexisting in many species. This colour polymorphism is likely under long‐term balancing selection, but the genetic and molecular mechanisms underlying the variation remain poorly understood. Here, we used transcriptome data alongside a novel chromosome‐level assembly to perform differential gene expression analysis in the club‐legged grasshopper *Gomphocerus sibiricus* (Caelifera: Acrididae: Gomphocerinae), aiming to identify the specific genes involved in the differentiation between green and brown morphs. Since differential expression analyses are prone to false positives, we replicated the analysis using an independent sample of individuals of the same species. We found six genes consistently upregulated in green individuals across both datasets, all annotated as beta‐carotene‐binding proteins (βCBPs). βCBPs are known to play a key role in the colour regulation in both the migratory locust *Locusta migratoria* and the desert locust *Schistocerca gregaria*, although their exact role may differ in the club‐legged grasshopper. The gene tree and chromosomal positions of βCBP copies in *G. sibiricus* , *L. migratoria* and *S. gregaria* indicate both ancestral (pre‐speciation) and lineage‐specific duplications. Our screening of publicly available orthopteran genomes revealed that homologues of the βCBP genes are largely absent from non‐Caelifera species when using conservative homology thresholds. This restricted distribution suggests that βCBP‐mediated pigmentation may represent a Caelifera‐specific mechanism that is involved in the production of green body coloration, while other orthopteran lineages likely rely on distinct genetic pathways. Together, our findings provide new insights and lay the groundwork for understanding the evolutionary diversification of pigmentation mechanisms in Orthoptera.

## 1 Introduction

The coexistence of multiple discrete colour phenotypes within local populations, known as colour polymorphism (Ford 1945; Huxley 1955; Forsman 2016; White and Kemp 2016), is a phylogenetically widespread phenomenon across animal and plant species (Leimar 2005). Colour polymorphism has attracted the interest of evolutionary biologists because it is relevant for understanding the evolutionary processes that maintain phenotypic diversity within populations (Gray and McKinnon 2007; McKinnon and Pierotti 2010; Svensson 2017). Furthermore, as a visual trait, body coloration allows researchers to study evolution in action in a way that can be easily traced in the field (Wright 1931, 1943). In some cases, phenotypically polymorphic states are transient and eventually fade back to monomorphism (Ford 1945, 1966; Mayr 1963; Forsman 2016). In others, morph frequencies appear to be stable and balanced in an equilibrium state (Mayr 1963; Forsman 2016). The causes of the persistence of phenotypic polymorphisms have been debated (Charlesworth et al. 1997), with balancing selection being the strongest candidate (Charlesworth 2006; Wellenreuther et al. 2014). Understanding how phenotypic polymorphisms arise and are maintained requires knowledge of the underlying genetic makeup of phenotypic polymorphisms. In most systems, however, the causal genetic variants have not yet been identified.

The co‐occurrence of colour morphs is quite common among arthropods (McKinnon and Pierotti 2010; Wellenreuther et al. 2014; Zhang et al. 2018) and has been studied in several polyneopteran insects (Forsman et al. 2002; Valverde and Schielzeth 2015; Tanaka et al. 2016). A particularly prominent example of a shared colour polymorphism is the green‐brown colour polymorphism in Orthoptera (crickets, bush crickets, grasshoppers and allies) (Rowell 1972; Dearn 1990), which represents one of the most widespread transspecies phenotypic polymorphisms in any group of organisms. This green‐brown polymorphism occurs in about 45% of the East African species of the family Acrididae (Rowell 1972) and in about 30% of the approximately 1050 European Orthoptera species (Schielzeth 2020). Remarkably, the green‐brown polymorphism is present in both major Orthoptera suborders, Ensifera and Caelifera, which diverged about 355 Mya (Song et al. 2020).

Insects typically display colour by absorbing or reflecting light through cuticular structures, pigments, or a combination of both (Chapman et al. 2013). Several classes of pigments contribute to insect coloration. Pterins, ommochromes, and carotenoids produce red, orange, and yellow hues, while melanins produce colours ranging from black to reddish brown (Fuzeau‐Braesch 1972). Additionally, flavonoids, which are secondary plant metabolites, can produce orange coloration in insects (Lindstedt et al. 2010). Green coloration in many insect orders, including Orthoptera, typically results from blue bile pigments (bilins) in combination with yellow carotenoids (Fuzeau‐Braesch 1972; Shamim et al. 2014; Varma et al. 2023). The bile pigments are likely located in the epidermal cells, while the yellow carotenoids are found in the cuticle or epidermis, together producing the characteristic green hue (Okay 1945, 1951, 1953; Fuzeau‐Braesch 1972; Shamim et al. 2014).

**FIGURE 1:**
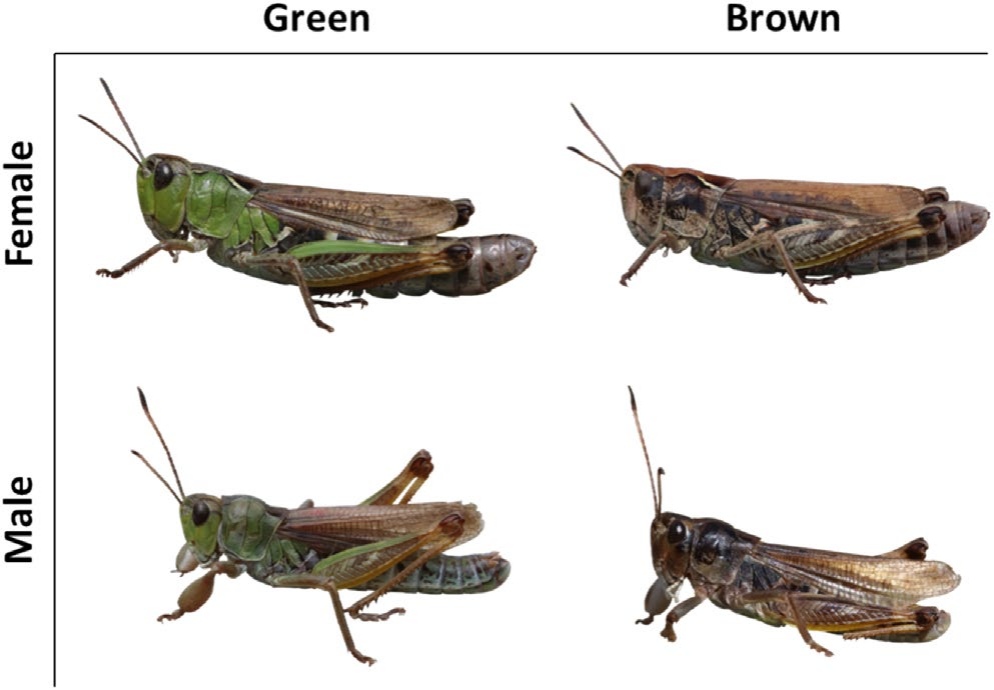
Distinct colour morphs in the club‐legged grasshopper *Gomphocerus sibiricus*.

We here focus on a species of gomphocerine grasshoppers within the Orthoptera, the club‐legged grasshopper *Gomphocerus sibiricus* (Caelifera: Acrididae: Gomphocerinae, Figure 1). Until recently, the factors influencing the green and brown colour morphs in gomphocerine grasshoppers were not well understood. Unlike some other Orthoptera, which are phenotypically plastic and can switch between green and brown colour morphs during development (often referred to as polyphenism) (Pener and Yerushalmi 1998; Pener and Simpson 2009), Gomphocerinae are characterised by genetic determination of the green‐brown polymorphism (phenotypic polymorphism *sensu stricta*) and insensitivity to environmental factors (Sansome and La Cour 1935; Gill 1981; Winter et al. 2021; Varma et al. 2025). Previous crossing experiments suggest that a small number of loci may be involved, with a putative green allele dominant over brown alleles (Schielzeth and Dieker 2020; Winter et al. 2021; Varma et al. 2025). It has been speculated that a functional genetic pathway is required for the formation of green and that this pathway is inactivated in brown individuals (Winter et al. 2021; Varma et al. 2025). Such a genetic basis probably applies to the club‐legged grasshopper (Schielzeth and Dieker 2020) and is likely to be shared among gomphocerine grasshopper species (and possibly beyond).

In this study, we analyse morph‐differential gene expression in the club‐legged grasshopper. As in other Gomphocerinae species, both sexes show green and brown morphs (Figure 1), with similar morph frequency biases in males and females (Schielzeth and Dieker 2020). We selected individuals from the last nymphal stage (N4) for RNA extraction and used transcriptomic analyses to identify differentially expressed genes (DEGs) between morphs. This approach provides a comprehensive analysis of gene expression profiles and sheds light on the pigments and molecular mechanisms underlying coloration. While DEGs may not directly correspond to loci harbouring sequence variation that controls colour morphs—since they can act downstream of causal loci or be regulated in trans—they provide valuable insight into which genes and regulatory pathways are involved in colour formation. A well‐known challenge of transcriptomic analyses is the potential for false positives, which may lead to spurious conclusions. To increase confidence in our results, we independently replicated the entire differential expression experiment to assess the reproducibility of candidate genes. We then investigated the evolution of the consistently differentially expressed genes identified in the two datasets by reconstructing gene trees based on homologues identified across three Acrididae species. This enabled us to examine patterns of gene diversification within the candidate gene family. To place these genes in a broader evolutionary context, we further screened their homologues across publicly available Orthopteran genomes and assessed their presence and divergence among related taxa.

## 2 Material and Methods

### 2.1 Sample Collection and Preparation

Club‐legged grasshoppers *Gomphocerus sibiricus* were collected in 2020 from a field site in the western Alps of France (45°4.8′N, 6°25.2′E). Only early instar nymphs of this species were collected. These were later reared in the laboratory until the fourth (last) nymphal stage (N4) (there are a total of four nymphal instars in this species). All individuals were reared in cages of 22 × 16 × 16 cm^3^ with *ad libitum* access to freshly cut grass in small water‐filled vials and a water tube for moisture. In the laboratory, club‐legged grasshoppers change between nymphal stages approximately every 5–7 days (shorter intervals for later stages). Approximately 2–3 days after moulting to the N4 stage, individuals were scored for colour morph and sex, then snap‐frozen and stored at −80°C for later RNA extraction.

### 2.2 Dissection and RNA Extraction

Accurate tissue selection and timing of sampling are critical for differential expression analyses. We selected individuals at the N4 stage for RNA extraction because colour differentiation begins at the second nymphal stage (N2), becomes distinct by the third nymphal stage (N3), and remains stable thereafter (Varma et al. 2023). By the N4 stage, colour morphs are fully developed, and no further colour changes occur during the transition to adulthood (own data and (Varma et al. 2023)). Sampling at the N4 stage increases the chance that functional transcripts, which directly contribute to body coloration, are maximally and stably expressed. It also minimises developmental variation across samples, allowing us to detect consistent morph‐associated differences in gene expression under controlled conditions.

We dissected and extracted RNA from 10 green (5 females, 5 males) and 12 brown (6 females, 6 males) N4 stage individuals. Half of the brown individuals represented the pied variant (Schielzeth and Dieker 2020), which is phenotypically distinct (mostly in females), but like the main brown morph is characterized by a lack of green. The inheritance pattern of the pied morphs is less well understood, and sample sizes are low, such that we focus our analysis on the comparison between green and brown (sensu lato, including pied) individuals. For each individual, the entire pronotum was dissected under a stereomicroscope using fine forceps and micro‐scissors. The underlying muscle tissue and any remaining internal organs, including parts of the digestive tract, were carefully removed to isolate the epidermal layer, where green and brown coloration is most pronounced. Particular care was taken to avoid contamination from deeper tissues or other body regions.

RNA extraction was performed from the cleaned epidermal tissue of the whole pronotum using the innuPREP RNA Mini Kit 2.0 (Analytik Jena), following the manufacturer's protocol. The resulting RNA pellet was resuspended in nuclease‐free water, and RNA quantity was measured using a NanoDrop spectrophotometer (Thermo Scientific). RNA integrity and purity were assessed with a Bioanalyzer (Agilent Technologies).

### 2.3 Illumina Sequencing

Library preparation and cDNA sequencing were outsourced to Biomarker Technologies (BMKGENE). Illumina sequencing generated a total of 519.97 million paired‐end (2 × 150 bp) reads (137.40 Gb) with an average of 20.88 million reads (6.25 Gb) per individual (range: 17.05–24.76 million reads, 5.10–7.40 Gb). Quality check was performed using FastQC v0.11.9 (Andrews 2010) and 91.54% of bases were above Q30. FastQC reported overrepresented sequences, which were identified via BLAST as mitochondrial transcripts from Orthoptera species. These sequences were retained, as they represent endogenous components of the transcriptome.

### 2.4 Confirmatory Dataset

Since differential gene expression analyses are prone to false positives even with multiple testing corrections, we replicated the entire analysis with an independent dataset. This dataset consisted of previously sequenced RNA samples from green and brown colour morphs of *G. sibiricus* collected in the Swiss Alps (46°31.0′N, 9°49.1′E) in 2017. It included 11 green individuals (4 females, 7 males) and 12 brown individuals (6 females, 6 males). Sequencing was performed by BGI Tech Solutions and generated a total of 1138.22 millions of paired‐end (2 × 100 bp) reads (113.822 Gb), with an average of 49.49 million reads (4.95 Gb) per individual (range: 40.47–62.35 million reads; 4.05–6.25 Gb). Throughout the manuscript, the samples from Switzerland will be referred to as the “Confirmatory Dataset” and the samples from France will be referred to as the “Discovery Dataset”. Note that although the confirmatory dataset also targeted the pronotum of N4 nymphs, tissue separation was less precise due to the occasional difficulty in isolating pure epidermal tissue from the underlying layers. As a result, RNA extracts from this dataset may contain transcripts from adjacent tissues. While this broader sampling does not compromise the identification of core morph‐specific expression differences, it increases the likelihood of detecting spurious expression signals from transcripts active in other tissue types.

### 2.5 Transcriptome Assembly and Differential Gene Expression Analysis

RNA‐seq reads from 22 individuals in the discovery dataset (average mapping rate: 97.3%) and 23 individuals in the confirmatory dataset (average mapping rate: 88.74%) were aligned to the recently generated *G. sibiricus* reference genome (Palacios‐Gimenez et al. 2025, DDBJ/ENA/GenBank accession JBPBLO000000000) using HISAT2 (v2.2.1) (Kim et al. 2019) with the flag *‐‐dta*. For each sample, transcript structures were annotated in GTF format using StringTie (v2.2.1) (Pertea et al. 2015). The resulting GTF files were then merged into a unified, non‐redundant transcript annotation using StringTie with the flag *‐‐merge*. Transcript sequences corresponding to the merged annotation were extracted from the genome using Gffread (v0.12.7) (Pertea and Pertea 2020). To reduce assembly noise and exclude likely fragmented or non‐functional transcripts, sequences shorter than 100 bp were removed.

The assembled transcriptome was used as a reference to quantify transcript expression with Salmon (v1.10.1) (Patro et al. 2017). Transcript‐level quantification was aggregated to the gene level expression and analysed using the DESeq2 R package (v1.38.3) (Love et al. 2014) for normalisation and differential expression analysis between the two morphs. While the genome‐based transcriptome assembly was constructed using individuals from both datasets, differential expression analyses were conducted separately for each dataset. Genes with an absolute log_2_ fold change (|log_2_FC|) > 2 and an adjusted *p*‐value (*p*_adj_) < 0.05 were considered as significantly differentially expressed. This stringent fold change threshold was chosen based on the expectation that functionally relevant transcripts would exhibit pronounced expression differences (potenitally even exclusive to one morph).

### 2.6 Annotation of Differentially Expressed Genes

Gene Ontology (GO) annotation of the significantly differentially expressed genes was performed using Blast2GO (version 6.0.3) (Conesa et al. 2005), with the longest transcript isoform for each gene used as the representative sequence. It turned out that all consistently differentially expressed genes share the same annotations (β‐carotene‐binding proteins, βCBPs, see result). To determine whether these differentially expressed genes truly represented distinct loci or alternatively spliced isoforms of a single gene, we aligned the corresponding transcript sequences to the *G. sibiricus* reference genome using Minimap2 (v2.26) (Li 2018) with setting *‐x splice:hq*. Furthermore, ORF for each of the transcripts belonging to the differentially expressed βCBP genes was predicted via NCBI's ORF Finder (www.ncbi.nlm.nih.gov/orffinder) and conserved domains in these sequences were identified using InterProScan (www.ebi.ac.uk/interpro/search/sequence).

### 2.7 Identification of Homologous Copies in Acrididae

Previous studies have implicated βCBPs in coloration in the desert locust *Schistocerca gregaria* (Acrididae: Cyrtacanthacridinae) (Egorkin, Dominnik, Maksimov, and Sluchanko 2024, Egorkin, Dominnik, Raevskii, et al. 2024) and the migratory locust *Locusta migratoria* (Acrididae: Oedipodinae) (Yang et al. 2019), supporting their potential role in pigmentation. To explore the evolution of this gene family, we searched for βCBP homologues across chromosome‐level genome assemblies of these two locust species and the newly chromosome‐level reference genome of the club‐legged grasshopper (Acrididae: Gomphocerinae). These lineages diverged approximately 45 Mya (Song et al. 2020).

The βCBP copies identified in the differential expression analysis were used as queries in a BLAST search (*E*‐value < 1e−10) against the predicted coding sequences (CDS) of *G. sibiricus* . Open reading frames (ORFs) were predicted for the resulting hits and translated into protein sequences. Proteins shorter than 100 amino acids were excluded, as the average length of βCBP proteins is approximately 250 amino acids. The remaining *G. sibiricus* protein sequences were then used as queries to identify homologues in the predicted proteomes of *S. gregaria* (NCBI accession: GCF_023897955.1) and *L. migratoria* (Li et al. 2024), using BLASTP (*E*‐value < 1e−10). For genes with multiple isoforms, the longest isoform was selected. Again, protein sequences shorter than 100 amino acids were excluded from further analysis. To identify additional βCBP copies in *G. sibiricus* , the homologues identified from *S. gregaria* and *L. migratoria* were used as queries to search against the *G. sibiricus* genome with MiniProt (version 0.13‐r248) (Li 2023).

### 2.8 Construction of βCBP Gene Tree

All resulting protein sequences were aligned using MUSCLE (as implemented in MEGA v11 (Edgar 2004)), and the alignment was refined with trimAl (v1.5.rev0) (Capella‐Gutiérrez et al. 2009) using the *‐automated1* setting to remove poorly aligned regions. A gene tree was constructed using MrBayes (v3.2.7) (Huelsenbeck and Ronquist 2001), with the WAG substitution model (*aamodelpr = fixed*(*wag*)) and gamma‐distributed rate variation among sites (*rates = gamma*). Two independent MCMC runs were conducted, each with four chains running for 1,000,000 generations and sampling every 100 generations. Convergence was assessed via the average standard deviation of split frequencies. The first 25% of sampled trees were discarded as burn‐in (*sumt burnin* = 250), and the remaining trees were used to summarise the posterior distribution of clades. Sequences that formed a distinct clade with the original βCBP candidates were considered as βCBP homologues for downstream analysis.

To investigate the evolutionary relationships among the identified βCBP homologues, we constructed a second gene tree using the same pipeline as described above: protein sequences were aligned using MUSCLE (as implemented in MEGA v11), trimmed with trimAl (*−automated1*), and the phylogeny was inferred using MrBayes (v3.2.7) under the WAG substitution model with gamma‐distributed rate variation (*aamodelpr* = *fixed*(*wag*), *rates* = *gamma*).

### 2.9 Identification of βCBP Distribution Across Orthoptera

To assess whether the βCBP genes are conserved in other Orthoptera species, we retrieved all available Orthoptera genome assemblies from NCBI (last accessed May 18, 2025). All βCBP homologues identified in previous analyses were aligned to these genomes using Miniprot (version 0.13‐r248) (Li 2023) with default parameters. To reduce false positives, only alignments that covered at least one‐third of the protein length were considered valid hits.

All tools were run with default parameters unless otherwise specified. Complete workflows, including all command‐line scripts, are available (see Data availability section).

## 3 Results

### 3.1 Genome‐Guided Transcriptome Assembly

We used RNA‐seq data from both the discovery and confirmatory datasets to construct a genome‐guided transcriptome assembly. In total, 83,761 transcripts corresponding to 51,080 genes were identified, with an N50 of 3688 bp (Table 1).

**TABLE 1:** Summary statistics of the genome‐guided transcriptome assembly.

Transcriptome assembly	Value
Total genes	51,080
Total transcripts	83,761
Percent GC	43.82
Contig N50 (bp)	3688
Contig length range (bp)	104–51,780
Average contig length (bp)	2140.76

### 3.2 Differentially Expressed Genes in the Discovery Dataset

**FIGURE 2:**
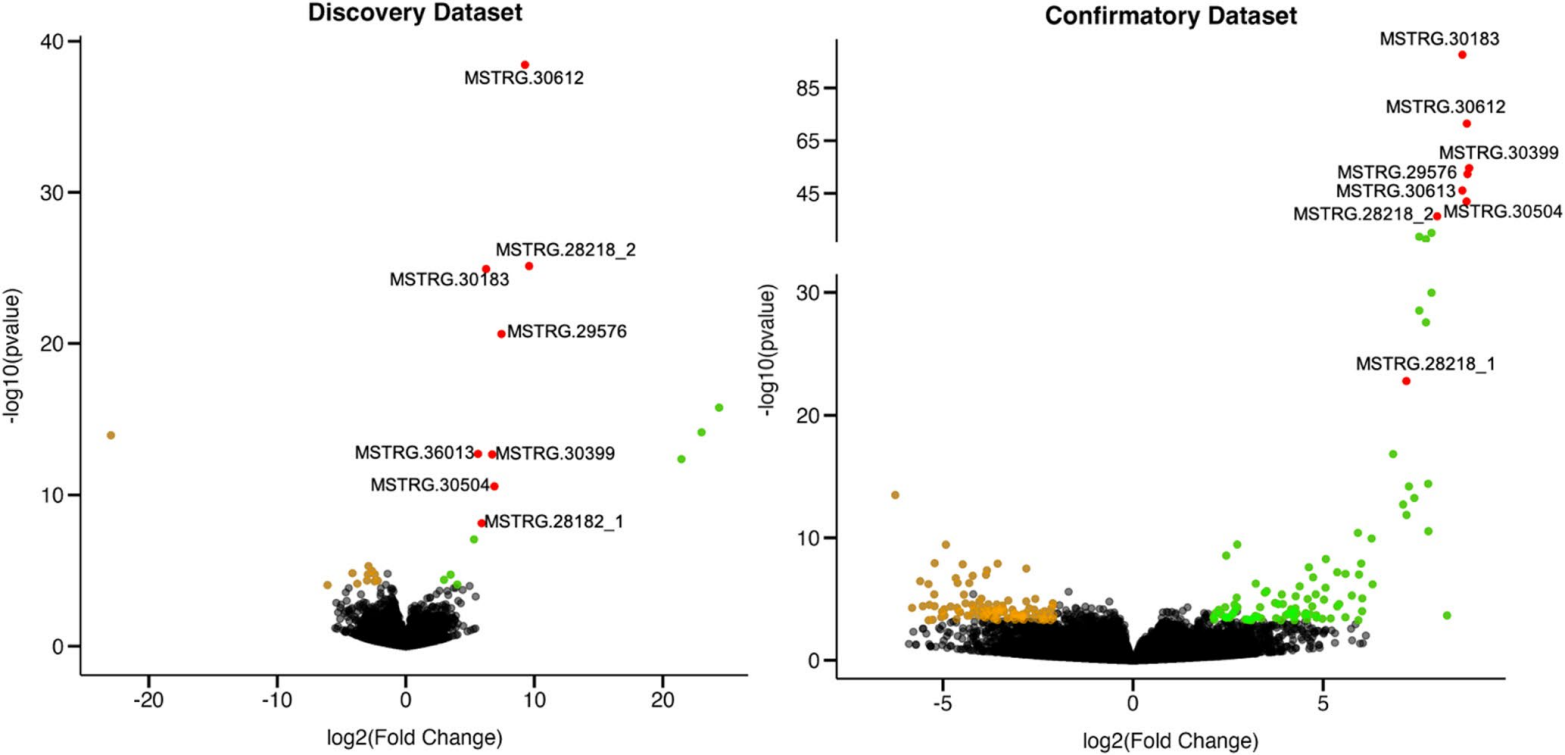
Differential gene expression in the club‐legged grasshopper *Gomphocerus sibiricus*. Each point in the volcano plot represents one gene. Green dots represent significantly upregulated genes (LFC > 2, *p*_adj_ < 0.05), brown dots represent significantly downregulated genes (LFC < −2, *p*_adj_ < 0.05). Red dots represent the overlapping upregulated genes between the discovery and confirmatory dataset. Isoforms of MSTRG.28218 were treated as two separated genes (MSTRG.28218_1 and MSTRG.28218_2).

Of the 30,802 genes with nonzero total read counts in the discovery dataset, 15,043 (49%) were excluded due to low mean expression by DESeq2's independent filtering process. Among the remaining 15,759 tested genes, 23 (0.15%) were identified as significantly differentially expressed (*p*_adj_ < 0.05 and |log_2_FC| > 2) between the two morphs. Of these, 13 genes were upregulated and 10 downregulated in the green morph (Figure 2; Table S1). Nine of the 23 genes were assigned at least one GO term (Table S2).

### 3.3 Differentially Expressed Genes in the Confirmatory Dataset

Of the 42,826 genes with nonzero total read counts in the confirmatory dataset, 19,040 (44%) were excluded due to low mean expression by DESeq2's independent filtering process. Among the 23,786 genes tested, 196 (0.82%) were significantly differentially expressed between morphs (*p*_adj_ < 0.05 and |log2FC| > 2). Ninety‐five genes were upregulated and 101 downregulated in green individuals (Figure 2 and Table S3). Given that tissue dissection in the confirmatory dataset was less precise, it is possible that some differentially expressed genes reflect contributions from mixed tissue types rather than true morph‐specific expression related to the colour phenotype. Sixty‐five of the 196 DEGs received GO annotations (Table S4).

### 3.4 Consistently Differentially Expressed Genes

Seven genes showed consistent upregulation in green morphs across both datasets (Table 2; Figure 2; Table S5; Figures S1 and S2), while no overlap was observed among the downregulated genes. Since 0.15% of genes were significantly differentially expressed in the discovery dataset and 0.82% in the confirmatory dataset, the probability of a random gene being significant in both datasets by chance is approximately *p* = 1 − (1 − 0.15%) × (1 − 0.82%)≈0.0097, or 0.97%. Furthermore, with a 50% probability of up‐ or downregulation by chance, the probability that a gene is upregulated in both datasets by chance is 0.5 × 0.5 = 25%. Therefore, the observation that seven genes were consistently upregulated in green individuals in both datasets is highly unlikely to occur by chance. These genes represent strong candidates associated with the regulation of the green–brown colour polymorphism in the club‐legged grasshopper.

**TABLE 2:** βCBP genes identified in differentially expression analysis.

Up regulated morph	Dataset	Gene ID	Transcript ID	Protein length	Chromosome/scaffold ID	Mapping position	Best Hit (accession No.)	βCBP No.
Green	Both	MSTRG.28218	g18974.t1	206	scaffold101	41	Takeout‐like protein 5 [AIY25506.1]	Gsib_scaffold101_1
			MSTRG.28218.3	111	scaffold101	47		—
			g18975.t1	276	scaffold101	484,272	Takeout‐like protein 5 [AIY25506.1]	Gsib_scaffold101_2
			MSTRG.28218.1	206	scaffold101	489,341		—
Green	Both	MSTRG.29576	g19169.t1	268	scaffold255	125,503	Putative beta‐carotene‐binding protein [XP_049787753.1]	Gsib_scaffold255_1
			MSTRG.29576.1	144	scaffold255	125,803		—
Green	Both	MSTRG.30399	g19331.t1	256	scaffold575	33,572	Putative beta‐carotene‐binding protein [XP_047118076.1]	Gsib_scaffold575_1
			MSTRG.30399.1	205	scaffold575	33,282		—
Green	Both	MSTRG.30612	g19390.t1	268	scaffold75	1,378,966	Putative beta‐carotene‐binding protein [XP_049787753.1]	Gsib_scaffold75_2
			MSTRG.30612.1	144	scaffold75	1,378,917		—
Green	Both	MSTRG.30613	g19392.t1	84	scaffold75	2,464,862	Putative beta‐carotene‐binding protein [XP_049816145.1]	—
Green	Both	MSTRG.30183	Gsib_scaffold469_ 000001.1	337	scaffold469	790	Putative beta‐carotene‐binding protein [XP_047000209.1]	—
			Gsib_scaffold469_ 000002.1	103	scaffold469	30,476		—
			MSTRG.30183.2	175	scaffold469	70		—
			MSTRG.30183.3	201	scaffold469	70		Gsib_scaffold469_1
Green	Both	MSTRG.30504	g19364.t1	62	scaffold657	17,125	Takeout‐like protein 5 [AIY25506.1]	—
Green	Confirmatory	MSTRG.1327	g965.t1	278	chr1	479,478,083	Putative beta‐carotene‐binding protein [XP_049832396.1]	Gsib_chr1_7
Green	Confirmatory	MSTRG.30609	Gsib_scaffold75_ 000003.1	261	scaffold75	104,568	Putative beta‐carotene‐binding protein [XP_049963929.1]	Gsib_scaffold75_1
Brown	Confirmatory	MSTRG.908	g642.t1	267	chr1	354,206,120	Putative beta‐carotene‐binding protein [XP_049815111.1]	Gsib_chr1_1
Brown	Confirmatory	MSTRG.8784	g5869.t1	268	chr2	685,747,996	Putative beta‐carotene‐binding protein [XP_049815111.1]	Gsib_chr2_1
			MSTRG.8784.1	233	chr2	685,747,919		—
			MSTRG.8784.2	203	chr2	685,747,927		—

*Note:* When multiple isoforms existed for a single gene, the longest isoform or the one with better alignment quality (higher identity and query coverage) was selected as the representative for that gene in downstream analysis. Isoforms of gene MSTRG.28218 were treated as two distinct genes. Two genes (MSTRG.30613 and MSTRG.30504) were excluded due to relatively short open reading frames. The column ‘βCBPs No.’ follows the nomenclature used in Tables 3 and 4, and Figures 3 and 4, in the format [species abbreviation]_[chromosome or scaffold ID]_[copy number]. For the log₂ fold change of each gene, please refer to the Table S5.

### 3.5 Analysis of Differentially Expressed Genes

All seven DEGs consistently upregulated in green morphs across both datasets were annotated as putative beta‐carotene‐binding proteins (βCBPs), based on sequence similarity to annotated βCBPs from *Schistocerca* species or to “takeout‐like protein 5” (an alternative name for βCBP; Egorkin, Dominnik, Raevskii, et al. 2024) from *Locusta migratoria* (Table 2). Alignment of these transcripts to the *Gomphocerus sibiricus* genome using Minimap2 revealed that they mapped to different genomic loci, suggesting they represent distinct genes rather than isoforms of a single gene (Table 2). Two of the seven DEGs (MSTRG.30613 and MSTRG.30504) encoded relatively short open reading frames (ORFs < 100 amino acids), and neither contained the hemolymph juvenile hormone‐binding protein (JHBP) domain typically found in other βCBP sequences (Table S5). These two DEGs may represent truncated transcripts, assembly artefacts, or non‐functional variants, and were thus excluded from the gene family analysis.

Transcript‐to‐genome alignments and reference genome annotations (GFF file) suggested that the four isoforms grouped under the gene ID MSTRG.28218 likely correspond to two distinct genes. Specifically, g18974.t1 and MSTRG.28218.3 appear to be isoforms of one βCBP gene (MSTRG.28218_1), while g18975.t1 and MSTRG.28218.1 appear to be isoforms of another (MSTRG.28218_2) (Table 2). We re‐ran the differential expression analysis treating these as two separate genes, and both remained significantly upregulated in green morphs across both datasets (Figure 2). We therefore treated them as distinct genes in the gene family analysis. In sum, we confidently identified six distinct βCBP genes that were consistently upregulated in green morphs.

In addition to the six βCBP genes consistently upregulated in green morphs across both datasets, four βCBP genes were differentially expressed only in the confirmatory dataset. Two of these (MSTRG.1327 and MSTRG.30609) were upregulated in green morphs, while two others (MSTRG.8784 and MSTRG.908) were downregulated (Table 2; Table S3; Figure S2). Although these genes were not identified as differentially expressed in the discovery dataset and might be false positives, we included them in the gene family analysis due to their sequence similarity to the consistently upregulated βCBPs.

### 3.6 Homologous Copies of βCBP in Acrididae

In total, 51, 42 and 34 potential βCBP homologues were identified in *G. sibiricus* , *S. gregaria* , and *L. migratoria*, respectively (Table S6). A gene tree constructed from these 127 protein sequences revealed that the 10 βCBP copies identified in the differential expression analysis form a distinct clade, together with nine additional βCBP copies from *G. sibiricus* , eight from *S. gregaria* and seven from *L. migratoria* (Figure S3 and Table 3). Most of these homologues showed consistent chromosomal localization across species. In *L. migratoria*, five of the seven βCBP genes were located on chromosome 5 (ranked by autosome size), and the remaining two on chromosome 2. Similarly, six of the eight *S. gregaria* copies were found on chromosome 5, with two located on chromosome 7. In *G. sibiricus* , seven of the 18 βCBP genes mapped to chromosome 1, two on chromosome 2, and one each on chromosomes 3 and 5. The remaining six were located on various unplaced scaffolds (Table 3 and Figure 4). These 33 sequences were considered high‐confidence βCBP homologues for subsequent analyses.

**TABLE 3:** βCBP copies used to build gene tree.

No.	Species	GeneID	Chromosome/scaffold ID	βCBP No.
1	*Schistocerca gregaria*	XP_049831452.1	chr5	Sgre_chr5_1
2	*Schistocerca gregaria*	XP_049832105.1	chr5	Sgre_chr5_2
3	*Schistocerca gregaria*	XP_049832104.1	chr5	Sgre_chr5_3
4	*Schistocerca gregaria*	XP_049831059.1	chr5	Sgre_chr5_4
5	*Schistocerca gregaria*	XP_049832205.1	chr5	Sgre_chr5_5
6	*Schistocerca gregaria*	XP_049832396.1	chr5	Sgre_chr5_6
7	*Schistocerca gregaria*	XP_049837939.1	chr7	Sgre_chr7_1
8	*Schistocerca gregaria*	XP_049837940.1	chr7	Sgre_chr7_2
1	*Gomphocerus sibiricus*	g642.t1	chr1	Gsib_chr1_1
2	*Gomphocerus sibiricus*	Gsib_chr1_005468.1	chr1	Gsib_chr1_2
3	*Gomphocerus sibiricus*	g644.t1	chr1	Gsib_chr1_3
4	*Gomphocerus sibiricus*	g645.t1	chr1	Gsib_chr1_4
5	*Gomphocerus sibiricus*	g647.t1	chr1	Gsib_chr1_5
6	*Gomphocerus sibiricus*	Gsib_chr1_005309.1	chr1	Gsib_chr1_6
7	*Gomphocerus sibiricus*	g965.t1	chr1	Gsib_chr1_7
8	*Gomphocerus sibiricus*	g5869.t1	chr2	Gsib_chr2_1
9	*Gomphocerus sibiricus*	Gsib_chr2_005186.1	chr2	Gsib_chr2_2
10	*Gomphocerus sibiricus*	g10412.t1	chr3	Gsib_chr3_1
11	*Gomphocerus sibiricus*	Gsib_chr5_000902.1	chr5	Gsib_chr5_1
12	*Gomphocerus sibiricus*	g18974.t1	scaffold101	Gsib_scaffold101_1
13	*Gomphocerus sibiricus*	g18975.t1	scaffold101	Gsib_scaffold101_2
14	*Gomphocerus sibiricus*	g19169.t1	scaffold255	Gsib_scaffold255_1
15	*Gomphocerus sibiricus*	MSTRG.30279.3	scaffold469	Gsib_scaffold469_1
16	*Gomphocerus sibiricus*	g19331.t1	scaffold575	Gsib_scaffold575_1
17	*Gomphocerus sibiricus*	Gsib_scaffold75_000003.1	scaffold75	Gsib_scaffold75_1
18	*Gomphocerus sibiricus*	g19390.t1	scaffold75	Gsib_scaffold75_2
1	*Locusta migratoria*	LOCMIG132150.1	chr5	Lmig_chr5_6
2	*Locusta migratoria*	LOCMIG133670.1	chr5	Lmig_chr5_5
3	*Locusta migratoria*	LOCMIG133680.1	chr5	Lmig_chr5_4
4	*Locusta migratoria*	LOCMIG133750.1	chr5	Lmig_chr5_2
5	*Locusta migratoria*	LOCMIG133780.1	chr5	Lmig_chr5_1
6	*Locusta migratoria*	LOCMIG035760.1	chr2	Lmig_chr2_1
7	*Locusta migratoria*	LOCMIG035770.1	chr2	Lmig_chr2_2

*Note:* The 33 βCBP copies identified in three Acrididae species that used to build the gene tree. The column ‘βCBPs No.’ follows the nomenclature used in Tables 2 and 4, and Figures 3 and 4, in the format [species abbreviation]_[chromosome or scaffold ID]_[copy number]. For the exact location of each copy, please refer to Table S6.

### 3.7 βCBP Gene Tree in Acrididae

**FIGURE 3:**
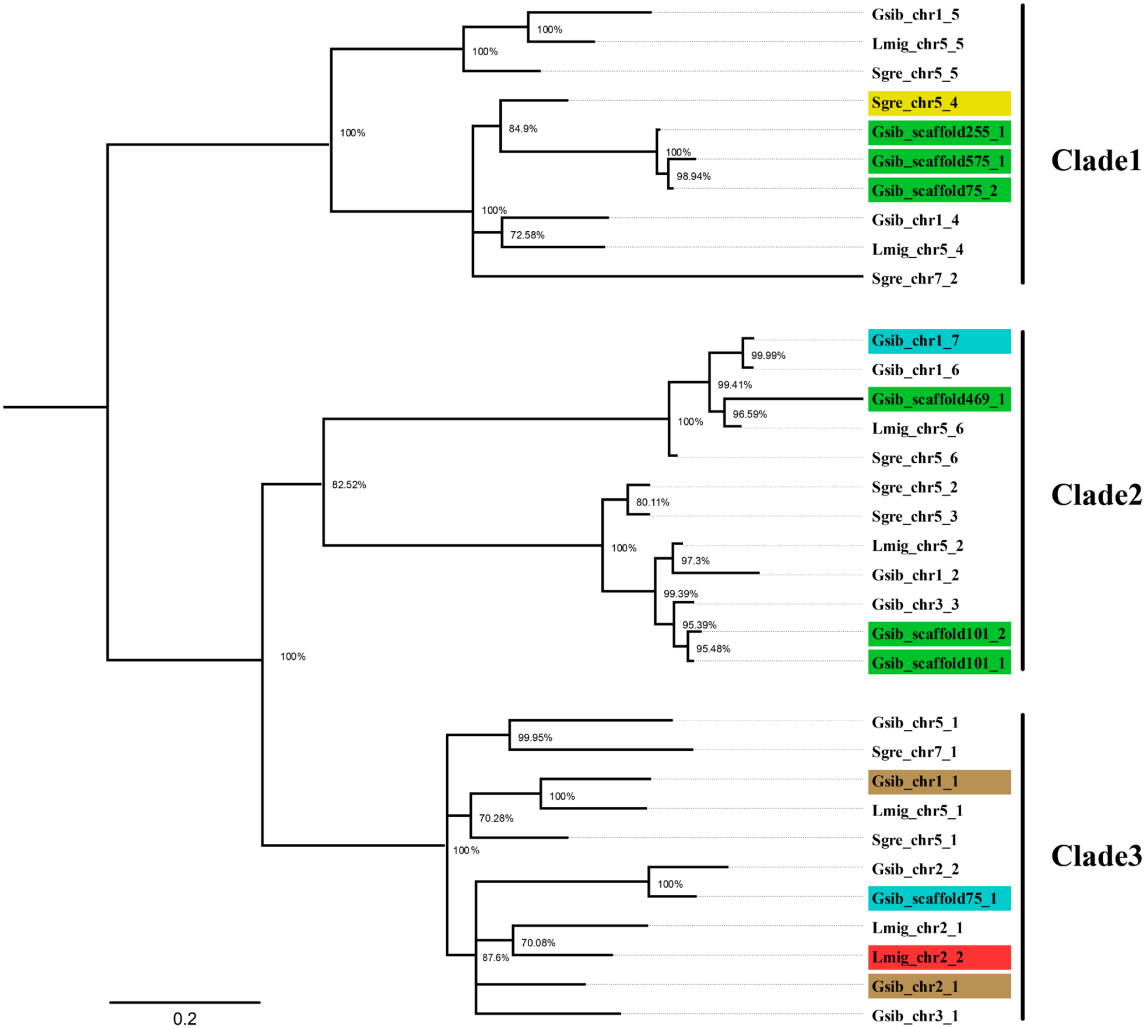
Gene tree of core βCBP gene copies across three Acrididae species. Gene names follow the format [species abbreviation]_[chromosome or scaffold ID]_[copy number], consistent with Figure 4, Tables 2 and 3. “Gsib” indicates *Gomphocerus sibiricus*, “Sgre” *Schistocerca gregaria*, and “Lmig” *Locusta migratoria*. Branch support values (posterior probabilities) are shown at each node as percentages. Branch lengths are drawn to scale; the scale bar represents 0.2 amino acid substitutions per site. The tree reveals three well‐supported clades. βCBP copies highlighted in green were consistently upregulated in green morphs across both discovery and confirmatory datasets of *G. sibiricus* . βCBP copies highlighted in blue (Gsib_scaffold75_1 and Gsib_chr1_7) were only upregulated in green morphs of confirmatory dataset of *G. sibiricus* . βCBP copies highlighted in brown (Gsib_chr2_1 and Gsib_chr1_1) were downregulated in brown morphs in the confirmatory dataset of *G. sibiricus* . The βCBP copy highlighted in yellow (Sgre_chr5_4) has been experimentally validated to bind β‐carotene and contribute to yellow coloration in *S. gregaria* . The βCBP copy highlighted in red (Lmig_chr2_2) is associated with red pigmentation during phase change in *L. migratoria*.

A gene tree constructed from the 33 high‐confidence βCBP homologues revealed three well‐supported clades (Figure 3). Of the 27 internal nodes, 20 had strong support with posterior probabilities ≥ 0.95. The six βCBP genes consistently upregulated in green morphs were distributed across Clade 1 and Clade 2. Specifically, three of the six copies formed a distinct subclade within Clade 1, together with a *S. gregaria* βCBP copy (Sgre_chr5_4), which has been experimentally shown to bind β‐carotene and contribute to yellow coloration in that species (Egorkin, Dominnik, Maksimov, and Sluchanko 2024, Egorkin, Dominnik, Raevskii, et al. 2024). In contrast, the two βCBP copies that were downregulated in green morphs in the confirmatory dataset were located in Clade 3. Of the two additional βCBP genes upregulated only in green morphs in the confirmatory dataset, one was found in Clade 2 and the other in Clade 3. The overall correspondence between expression patterns and phylogenetic clades suggests possible subfunctionalization among *G. sibiricus* βCBP paralogues.

### 3.8 Taxonomic Distribution of βCBP in Orthoptera

To investigate the broader taxonomic distribution of βCBP genes, we assessed the distribution of βCBP genes across publicly available Orthoptera genomes on NCBI (Table S7). Among the 23 assemblies examined (excluding one labelled as ‘contaminated’), homologues of the 18 *G. sibiricus* βCBP protein sequences were consistently detected in *L. migratoria* and multiple *Schistocerca* species (Table 4). Additionally, eight homologues were identified in *Vandiemenella viatica*. These results were based on a conservative filtering criterion requiring hits to cover at least one‐third of the query protein length. When no coverage filter was applied, additional matches were observed in several other Orthoptera species, indicating a potentially broader, but more divergent, distribution of βCBP‐like sequences across the order (Table 4). Similar distribution patterns were observed when βCBP homologues from *S. gregaria* and *L. migratoria* were used as queries (Table S7 and Table S8), though two Ensifera species each yielded a single hit meeting the one‐third query length criterion with *S. gregaria* homologues, and these hits corresponded to different βCBP copies within *S. gregaria* .

**TABLE 4:** Presence of *Gomphocerus sibiricus* βCBP homologues across Orthopteran species.

Suborder	Family	Species	1	2	3	4	5	6	7	8	9	10	11	12	13	14	15	16	17	18
Caelifera	Acrididae	*Locusta migratoria*	**	**	**	**	**	**	**	**	**	**	**	**	**	**	**	**	**	**
	Acrididae	*Schistocerca americana*	**	**	**	**	**	**	**	**	**	**	**	**	**	**	**	**	**	**
	Acrididae	*Schistocerca cancellata*	**	**	**	**	**	**	**	**	**	**	**	**	**	**	**	**	**	**
	Acrididae	*Schistocerca gregaria*	**	**	**	**	**	**	**	**	**	**	**	**	**	**	**	**	**	**
	Acrididae	*Schistocerca nitens*	**	**	**	**	**	**	**	**	**	**	**	**	**	**	**	**	**	**
	Acrididae	*Schistocerca piceifrons*	**	**	**	**	**	**	**	**	**	**	**	**	**	**	**	**	**	**
	Acrididae	*Schistocerca serialis_cubense*	**	**	**	**	**	**	**	**	**	**	**	**	**	**	**	**	**	**
	Morabidae	*Vandiemenella viatica*	**			**	**				**	**				**		**		**
	Tetrigidae	*Tetrix japonica*																		
	Tetrigidae	*Eucriotettix oculatus*																		
	Tetrigidae	*Zhengitettix transpicula*			*															
Ensifera	Gryllidae	*Acheta domesticus*							*							*				*
	Gryllidae	*Gryllodes sigillatus*						*								*				
	Gryllidae	*Gryllus assimilis*							*											
	Gryllidae	*Gryllus bimaculatus*						*												
	Gryllidae	*Gryllus longicercus*																		
	Gryllidae	*Myrmecophilus myrmecophilus*		*																
	Gryllidae	*Teleogryllus occipitalis*															*			
	Gryllidae	*Teleogryllus oceanicus*				*														
	Gryllotalpidae	*Gryllotalpa orientalis*		*																
	Tettigoniidae	*Anabrus simplex*			*									*						
	Tettigoniidae	*Meconema thalassinum*		*																
	Trigonidiidae	*Laupala kohalensis*																		

*Note:* ‘**’ indicates homologues detected after applying the filter criterion (coverage length > 1/3 of the query length), while ‘*’ indicates homologues detected without applying the filter. Number on the first row indicates different homologues of *G. sibiricus* , with the same order list in Table 3. Please refer to Table S7 for the exact accession number for each species genome assembly.

## 4 Discussion

Our study aimed to uncover genes mediating the green–brown polymorphism in the club‐legged grasshopper *Gomphocerus sibiricus* by identifying those differentially expressed between the green and brown morphs. Characterisation of these genetic differences is critical for understanding the molecular mechanisms that control morph‐specific coloration and lays the foundation for future pigmentation studies within the Gomphocerinae and Orthoptera more broadly. We identified six beta‐carotene‐binding protein (βCBP) genes, each located at distinct genomic positions, that are significantly and consistently upregulated in green morphs across two independent datasets. This strongly implicates that members of the βCBP gene family are key mediators of colour variation in the club‐legged grasshopper. Our findings thus advance knowledge of the molecular basis of colour polymorphism in orthopterans and provide a framework for further evolutionary and ecological investigations.

The green coloration in orthopterans is believed to result from a combination of yellow carotenoids and blue biliverdin (Okay 1945, 1951, 1953; Fuzeau‐Braesch 1972; Shamim et al. 2014). Given the key role of carotenoids in coloration, proteins that bind and transport carotenoids, such as βCBP, are potential mediators of colour expression. In the desert locust *Schistocerca gregaria*, βCBP facilitates carotenoid uptake and transport, resulting in yellow body coloration (Egorkin, Dominnik, Maksimov, and Sluchanko 2024, Egorkin, Dominnik, Raevskii, et al. 2024). In the migratory locust *Locusta migratoria*, βCBP modulates carotenoid dynamics to regulate pigmentation (Yang et al. 2019). Furthermore, in the silkworm *Bombyx mori* , the functional form of cellular carotenoid binding proteins, which bind to both lutein and β‐carotene, are essential for yellow cocoon coloration, whereas mutations in this gene result in colourless hemolymph and white cocoons (Tabunoki et al. 2004; Sakudoh et al. 2007). These studies highlight the role of βCBP in insect pigmentation.

Based on the previous studies in other insects and our own results, we hypothesize that βCBPs facilitate carotenoid uptake and transport in the club‐legged grasshopper. Specifically, we propose that upregulation of βCBPs in green morphs promotes the formation of a βCBP–beta‐carotenoid complex that generates a yellow pigment. Combined with biliverdin, this contributes to the green coloration characteristic of the green morphs. Supporting this hypothesis of two‐components coloration, shed skins from green nymphs exhibit a yellowish hue, whereas those from brown nymphs appear colourless (Varma et al. 2023). This suggests that the yellow βCBP–beta‐carotenoid complex accumulates in the cuticle (Egorkin, Dominnik, Maksimov, and Sluchanko 2024, Egorkin, Dominnik, Raevskii, et al. 2024) and in a morph‐specific manner. Further support comes from our gene tree analysis (Figure 3), where three βCBP paralogues consistently upregulated in green morphs cluster with the *S. gregaria* βCBP (Sgre_chr5_4), which has been experimentally shown to bind β‐carotene and contribute to yellow pigmentation (Egorkin, Dominnik, Maksimov, and Sluchanko 2024, Egorkin, Dominnik, Raevskii, et al. 2024). This phylogenetic clustering suggests a conserved carotenoid‐binding function among certain βCBP copies within Acrididae. We note that although not all Acrididae species exhibit within‐species colour polymorphism, βCBP genes generally contribute to green coloration, and differential regulation of these genes may underlie green–brown polymorphisms in some species.

Interestingly, two genes that were downregulated in the green morphs of the confirmatory dataset were also annotated as βCBP (Table 2). They were not detected in the discovery dataset, indicating they may be false positives. In any case, the gene tree analysis revealed that these two copies (Gsib_chr1_1 and Gsib_chr2_1) form a separate clade (Clade 3) from the βCBPs that were consistently upregulated in green morphs (Figure 3). This distinct grouping and opposing expression of βCBP paralogues point to possible subfunctionalization.

**FIGURE 4:**
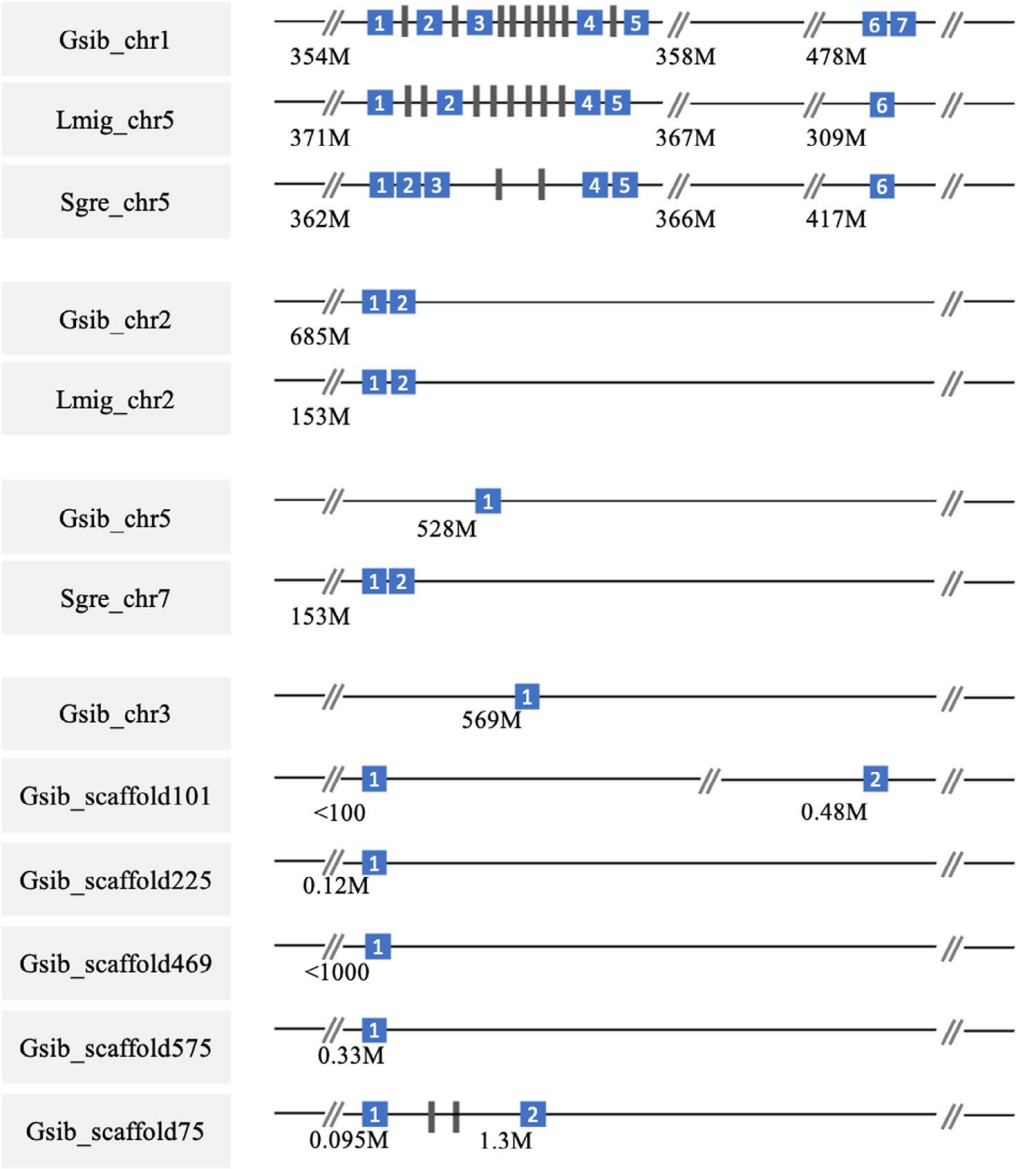
Schematic representation of the genomic location of βCBP copies in three Acrididae species. Each line represents a chromosome or scaffold, with names shown in the left column. These names correspond to those used in Figure 3 and follow the format [species abbreviation]_[chromosome or scaffold ID]_[copy number]. “Gsib” indicates *Gomphocerus sibiricus*, “Sgre” *Schistocerca gregaria*, and “Lmig” *Locusta migratoria*. Blue squares with numbers indicate βCBP copies found on that chromosome or scaffold. Grey bars represent genes located between βCBP copies. Numbers below each chromosome or scaffold indicate the approximate position of the nearest βCBP copy. Note that this diagram illustrates the relative locations of βCBP copies, not their exact genomic positions. The orientation of Lmig_chr5 is reversed relative to the others.

In the migratory locust (*L. migratoria*), βCBP–beta‐carotenoid complexes are involved in phase‐dependent pigmentation changes. During gregarization, a specific βCBP is upregulated, promoting the deposition of a red pigment complex on a green background, which contributes to the darker appearance of gregarious individuals (Yang et al. 2019). Based on genome alignment, this βCBP maps to chromosome 2 and corresponds to Lmig_chr2_2 in our gene tree (Figures 3 and 4; Table S9), which clusters with the two βCBPs downregulated in green morphs of *G. sibiricus* (Clade 3), suggesting potential functional similarity. Thus, the reversal of pigmentation outcomes—where βCBP upregulation leads to darkening in *L. migratoria* (via red pigment deposition on green) but to green coloration in *G. sibiricus* (via formation of a yellow βCBP–beta‐carotenoid complex)—again underscores the subfunctionalization of βCBP paralogues and highlights their lineage‐specific roles in pigmentation.

The other βCBP copy on chromosome 2 of *L. migratoria* (Lmig_chr2_1), which was not significantly differentially expressed between gregarious and solitary individuals, was associated with green coloration (Yang et al. 2019). This copy also falls within Clade 3 of our gene tree, a clade that generally groups βCBP copies not associated with green pigmentation. Intriguingly, one *G. sibiricus* copy (Gsib_scaffold75_1), upregulated in green morphs in the confirmatory dataset, clusters within the same subclade (Figure 3). However, since this copy was not recovered in the discovery dataset, it may represent a false positive. Its closest *G. sibiricus* paralogue (Gsib_chr2_2) resides on chromosome 2. Given the high chromosome synteny between *G. sibiricus* and *L. migratoria* (Palacios‐Gimenez et al. 2025), see below, these chromosome 2 copies may represent conserved homologues, although the local genomic context around these genes is rather complex. Specifically, Gsib_chr2_1—upregulated in brown morphs—appears homologous to Lmig_chr2_2, which promotes red pigmentation; Gsib_chr2_2—whose closest paralog (Gsib_scaffold75_1) upregulated in green morphs in the confirmatory dataset—may correspond to Lmig_chr2_1, previously implicated in green coloration in *L. migratoria* (Yang et al. 2019). The presence of green‐associated copies within Clade 3 may result from limited resolution within this clade, which obscures finer‐scale phylogenetic structure. The apparent inconsistency might reflect unresolved relationships among closely related paralogues, rather than true functional convergence or misannotation.

The chromosome synteny among *G. sibiricus* , *L. migratoria*, and *S. gregaria* is generally high (Palacios‐Gimenez et al. 2025), but the (European) Gomphocerinae lineage features three centric fusions: between chromosomes 1 and 5, 2 and 6, and 3 and 8 (as ranked by autosome size in *L. migratoria* and *S. gregaria* ). These fused pairs correspond to chromosomes 1, 2, and 3 in *G. sibiricus* (Palacios‐Gimenez et al. 2025). The chromosomal locations of βCBP genes in *G. sibiricus* align with these fusions and show strong correspondence with phylogenetic clades. In particular, the βCBP genes located on chromosome 1 in *G. sibiricus* align closely with their homologues on chromosome 5 in the other two species. For example, Gsib_chr1_1 clustered with Sgre_chr5_1 and Lmig_chr5_1, Gsib_chr1_2 with Sgre_chr5_2 and Lmig_chr5_2, and so on—both in genomic position and in gene tree topology (Figures 3 and 4). Notably, *L. migratoria* appears to lack a gene corresponding to copy number 3, which is present in both *G. sibiricus* and *S. gregaria* . This suggests a possible lineage‐specific gene loss in *L. migratoria* (we accounted for this potential deletion in our numbering in Figures 3 and 4). Similar patterns emerge on other chromosomes. The βCBP copies located on chromosome 2 of *G. sibiricus* cluster with their counterpart on chromosome 2 of *L. migratoria* (see above). Likewise, the copy on Gsib_chr5 clusters with Sgre_chr7_2, and these two chromosomes also show conserved synteny (Palacios‐Gimenez et al. 2025). Together, these patterns indicate that conserved synteny has maintained a strong correspondence between gene phylogeny and chromosomal location across these species.

The phylogenetic relationships and genomic positions of βCBP copies further suggest that both ancestral (pre‐speciation) and lineage‐specific duplications have shaped the evolution of this gene family. In particular, the βCBP copies located on chromosome 5 of *S. gregaria* likely represent ancient duplications that occurred before the divergence of the three species, as indicated by their conserved synteny and phylogenetic clustering across species (Figures 3 and 4). Interestingly, one βCBP copy on chromosome 7 of *S. gregaria* (Sgre_chr7_2) clusters with homologues on chromosome 5 (Sgre_chr5) rather than with its chromosomal neighbour Sgre_chr7_1, suggesting it may have originated from a duplication of a βCBP on chromosome 5. Supporting this, we found that genes next to Sgre_chr7_2 show high sequence similarity to those flanking Sgre_chr5_4 and Sgre_chr5_1 (Table S10).

Beyond these ancestral copies, *G. sibiricus* shows additional lineage‐specific expansion of the βCBP gene family. The comparatively high number of βCBP copies in *G. sibiricus* may be linked to its genome's elevated transposable element (TE) content (~82%; Palacios‐Gimenez et al. 2025). Indeed, several scaffolds harboring βCBP genes contain few annotated genes and are largely composed of repetitive DNA sequences. These TEs not only complicate the assignment of such scaffolds to specific chromosomes, but also raise the possibility that some βCBP duplications may have arisen through TE‐mediated transposition, promoting diversification (Cabral‐de‐Mello and Palacios‐Gimenez 2025).

Our genomic screening of βCBP homologues across Orthoptera revealed their presence in species from 11 different subfamilies beyond Gomphocerinae. However, when applying a conservative alignment coverage threshold (i.e., requiring homologues to match at least one‐third of the query length), only a smaller set of confident hits remained (Table 4). These were primarily restricted to species within the family Acrididae and *Vandiemenella viatica* (Caelifera: Morabidae). This phylogenetic distribution suggests that βCBP genes associated with pigmentation have undergone specialisation and diversification within Caelifera, particularly in Acrididae, while homologues in other lineages are likely too divergent to be detected under stringent criteria. The presence of βCBP‐like sequences in *V. viatica* suggests that the gene family has been conserved within Caelifera for at least approximately 200 million years, as Morabidae (Eumastacoidea) and Acrididae (Acridoidea) diverged around that time (Song et al. 2015, 2020). However, further investigation is needed to clarify the functional conservation and evolutionary trajectories of these genes across lineages.

The apparent restriction of pigmentation‐related βCBP genes to Caelifera suggested that these genes may have evolved a clade‐specific role in colour manifestation. However, green–brown polymorphisms also occur in non‐Caeliferan species such as *Anabrus simplex* (Tettigoniidae), where we did not detect any clear βCBP homologues. This suggests that similar phenotypes, such as green coloration, may have evolved independently in different orthopteran lineages through distinct genetic mechanisms, highlighting the presence of multiple evolutionary solutions to the similar ecological or signalling challenge.

A recent study (Egorkin et al. 2025) demonstrated that green coloration in *Tettigonia cantans* (Ensifera: Tettigoniidae: Tettigoniinae) is produced by a dichromophoric protein capable of binding both luteins (yellow) and bilins (blue), thereby producing a green pigment through simultaneous chromophore binding. While this mechanism is unlikely to be universal, it appears to be common within Ensifera (Egorkin et al. 2025). By contrast, Caeliferan species, including those in Acrididae, seem to rely on two distinct proteins—one binding carotenoids (e.g., βCBP) and another binding biliverdin (e.g., BBP)—to achieve green coloration through pigment layering or mixing (Egorkin et al. 2025). This functional divergence may explain why Ensiferan species in our screening lacked detectable βCBP homologues, while these genes were more reliably identified in Caeliferans. Further comparative genomic analyses and broader taxonomic sampling will be crucial to reconstruct the evolutionary history of βCBP genes and to clarify their roles in pigmentation across Orthoptera.

To our knowledge, this is the first study to demonstrate the role of βCBP in pigmentation within Gomphocerinae grasshopper species, thereby extending the molecular understanding of green–brown polymorphism beyond locust species. Previous genetic studies suggest that a shared genetic basis underlies this polymorphism within Gomphocerinae (Schielzeth and Dieker 2020; Winter et al. 2021; Varma et al. 2025). Given the observed Mendelian inheritance patterns, it is plausible that βCBPs function as downstream mediators in conserved pigmentation pathways. However, this hypothesis remains to be tested in Gomphocerinae and in non‐locust species showing phenotypic plasticity in coloration, where colour expression may be influenced by additional molecular or environmental cues.

Future work should focus on functionally validating the role of βCBP in pigmentation. Targeted approaches such as RNA interference (RNAi) or CRISPR‐Cas9‐mediated knockouts can directly assess whether changes in βCBP expression impact the carotenoid–biliverdin balance and thereby alter body coloration. Dissecting the regulatory mechanisms controlling βCBP expression, such as cis‐regulatory elements or trans‐acting factors, will clarify how expression is modulated across morphs. Expanding comparative analyses to a broader range of Orthoptera will further reveal whether βCBP plays a conserved or lineage‐specific role in pigmentation. Moreover, the evidence for subfunctionalization among βCBP copies highlights the need to explore functional divergence within this gene family and its broader physiological roles beyond pigmentation.

Our identification of βCBPs as key candidate genes in the regulation of green–brown polymorphism provides a molecular entry point to address fundamental ecological and evolutionary questions. Such polymorphisms are often subject to natural selection due to their potential influence on crypsis, thermoregulation (Köhler and Schielzeth 2020; Cabon and Schielzeth 2025), and habitat use (Heinze et al. 2022), which can lead to differential survival and reproductive success under varying environmental conditions. Understanding the genetic basis of colour morphs enables us to examine how selection shapes phenotypic diversity in wild populations. The persistence of both green and brown morphs may be maintained by spatial or temporal environmental heterogeneity (Dieker et al. 2018), a hypothesis that can be tested once the genomic variants influencing βCBP expression are identified. Integrating molecular data with field‐based studies of morph‐specific fitness, behaviour, and ecological interactions will be essential to uncover the mechanisms maintaining this polymorphism.

## Author Contributions

Conceptualization: H.S.; data collection: M.V., A.S.; data curation: M.V., C.J., A.S., O.M.P.‐G., H.S.; formal analysis: C.J., M.V., A.S., H.S.; funding acquisition: H.S.; writing – original draft: M.V., C.J.; writing – review and editing: M.V., C.J., A.S., O.M.P.‐G., H.S.

## Conflicts of Interest

The authors declare no conflicts of interest.

## Supporting information


**Figure S1:** Raw counts of the consistently differently expressed genes in discovery dataset. Transcripts of gene MSTRG.28218 were treated as two separated genes (MSTRG.28218_1 and MSTRG.28218_2).
**Figure S2:** Raw counts of the differently expressed genes in confirmatory dataset. (a) Raw counts of the consistently differently expressed genes; (b) differently expressed genes only found in confirmatory dataset.
**Figure S3:** Gene tree of all putative βCBP copies across three Acrididae species. Gene names starting with “XP_” are copies from *Schistocerca gregaria*; names starting with “LOCMIG” are from *Locusta migratoria*; all remaining copies are from *Gomphocerus sibiricus*. These gene copies are also listed in Table S6. Copies highlighted in red rectangle were used in to build the second gene tree.

**Data S1:** mec70142‐sup‐0002‐DataS1.xlsx.

## Data Availability

Sequence data have been deposited in the NCBI Sequence Read Archive (BioProject PRJNA1241690). Data and code are available at https://doi.org/10.5061/dryad.qz612jmv2.
