# Supplementary material for: Replicated Differential Expression Analysis in a Green‐Brown Polymorphic Grasshopper Reveals Role of Beta‐Carotene‐Binding Protein in Body Coloration
Source: Mol Ecol. 2025 Oct 22;34(22):e70142. doi: 10.1111/mec.70142 (PMC12617024; doi:10.1111/mec.70142)
Supplement: Supplementary file 1 — Figure S1: Raw counts of the consistently differently expressed genes in discovery dataset. Transcripts of gene MSTRG.28218 were treated as two separated genes (MSTRG.28218_1 and MSTRG.28218_2). Figure S2: Raw counts of the differently expressed genes in confirmatory dataset. (a) Raw counts of the consistently differently expressed genes; (b) differently expressed genes only found in confirmatory dataset. Figure S3: Gene tree of all putative βCBP copies across three Acrididae species. Gene names starting with “XP_” are copies from Schistocerca gregaria; names starting with “LOCMIG” are from Locusta migratoria; all remaining copies are from Gomphocerus sibiricus. These gene copies are also listed in Table S6. Copies highlighted in red rectangle were used in to build the second gene tree. [file MEC-34-e70142-s001.pdf]

Figure S1 Raw counts of the consistently differently expressed genes in discovery dataset. Transcripts of gene MSTRG.28218 were treated as two separated genes (MSTRG.28218_1 and MSTRG.28218_2).

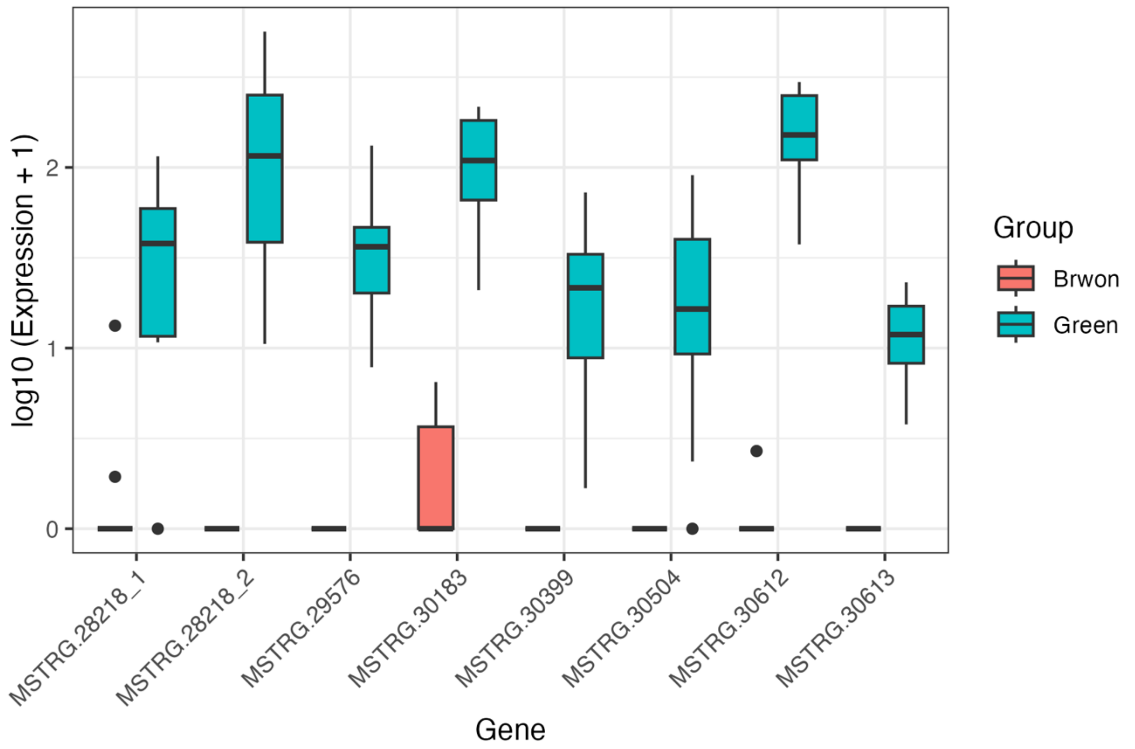

Figure S2 Raw counts of the differently expressed genes in confirmatory dataset**.** (a) Raw counts of the consistently differently expressed genes; (b) differently expressed genes only found in confirmatory dataset.

(a)

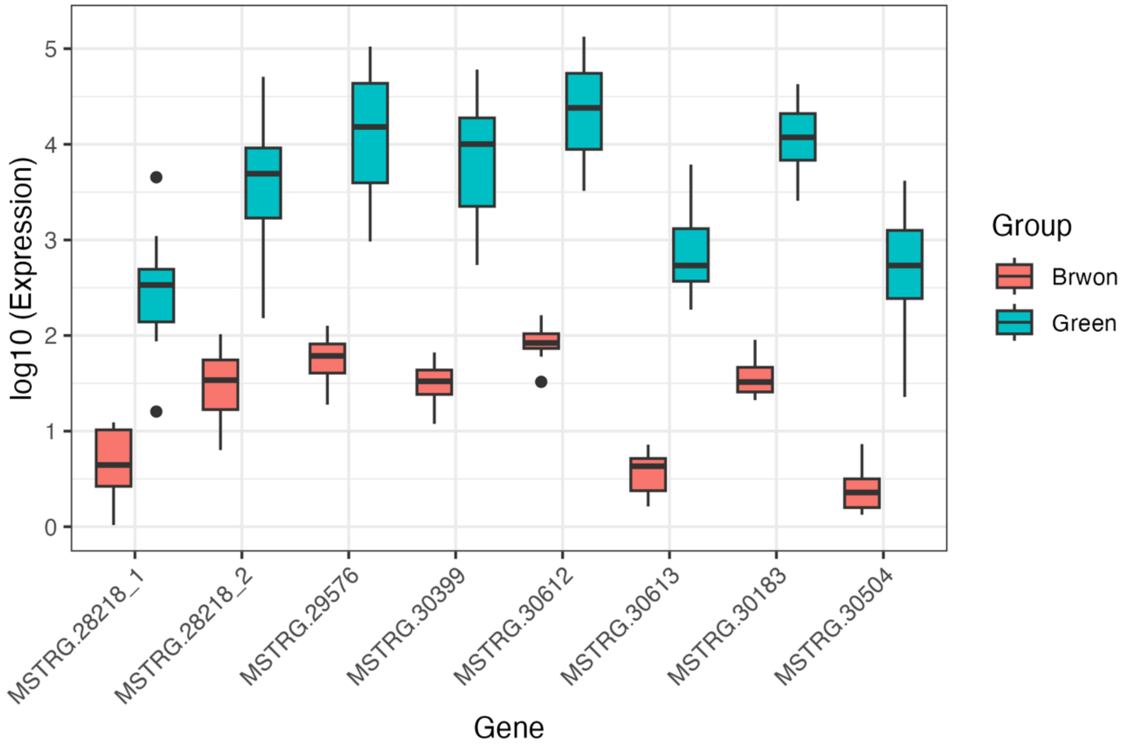

(b)

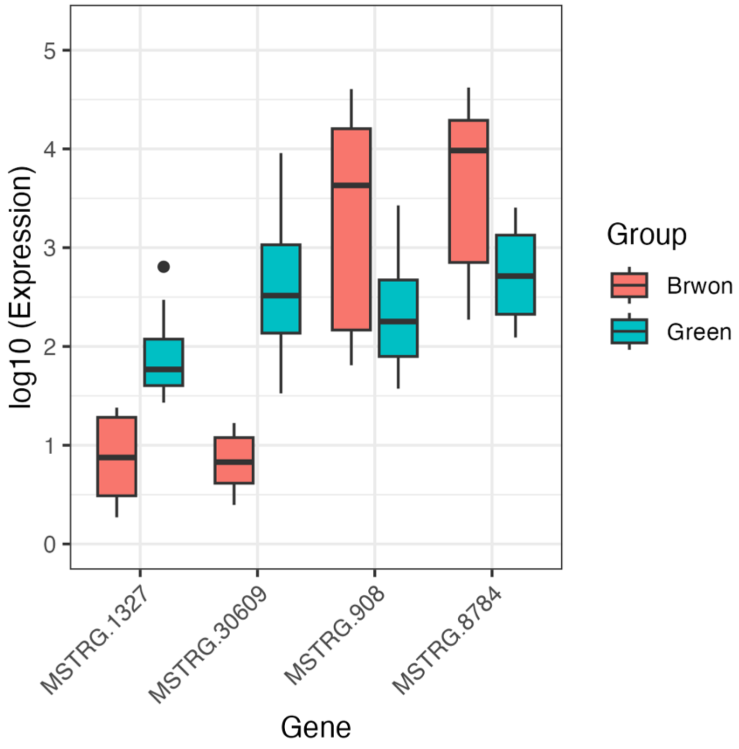

Figure S3 Gene tree of all putative βCBP copies across three Acrididae species. Gene names starting with "XP_" are copies from *Schistocerca gregaria*; names starting with "LOCMIG" are from *Locusta migratoria*; all remaining copies are from *Gomphocerus sibiricus*. These gene copies are also listed in Table S6. Copies highlighted in red rectangle were used in to build the second gene tree.

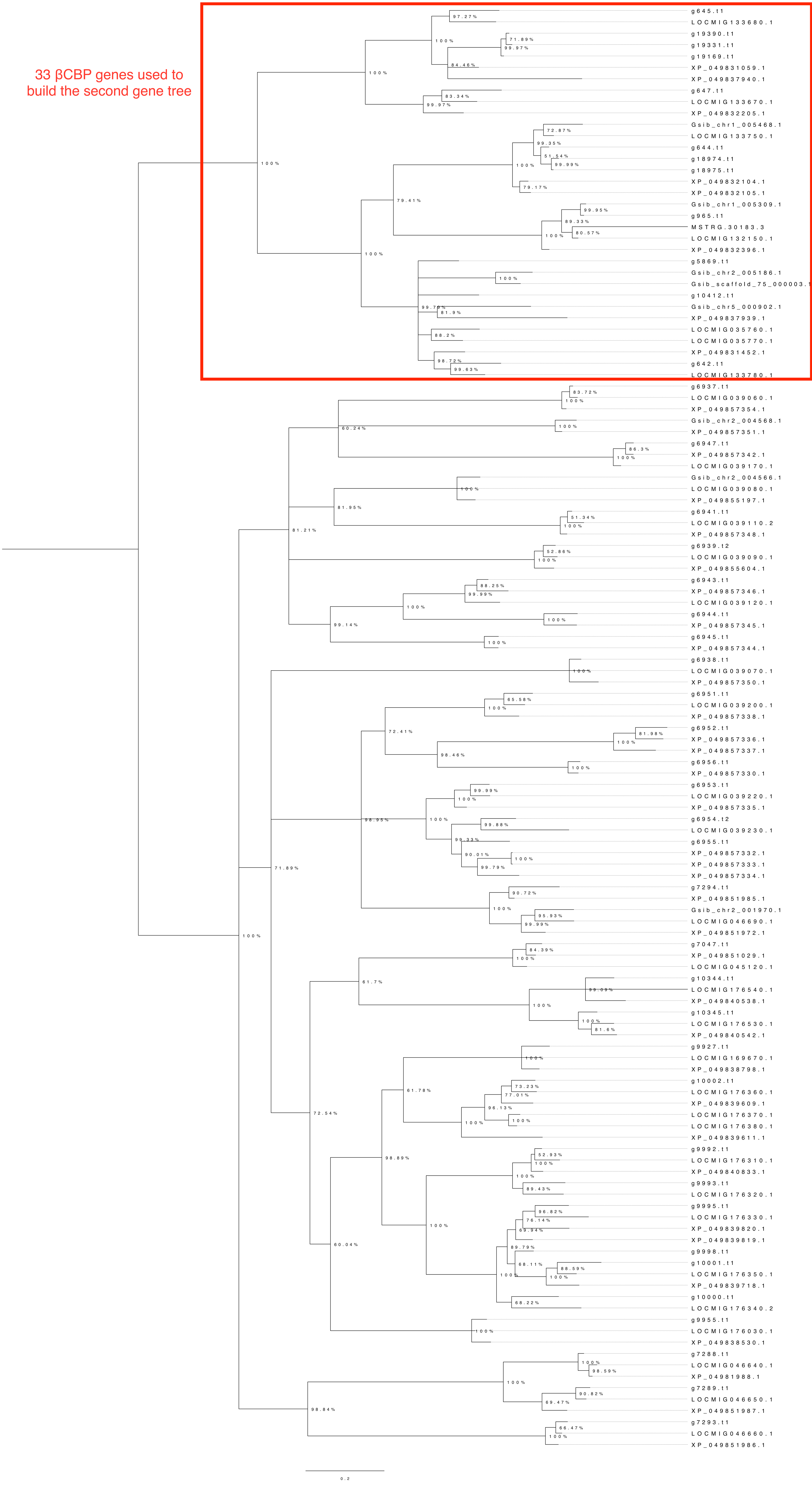
33 βCBP genes used to build the second gene tree
g645.t1
LOCMIG133680.1
g19390.t1
g19331.t1
g19169.t1
XP_049831059.1
XP_049837940.1
g647.t1
LOCMIG133670.1
XP_049832205.1
Gsib_chr1_005468.1
LOCMIG133750.1
g644.t1
g18974.t1
g18975.t1
XP_049832104.1
XP_049832105.1
Gsib_chr1_005309.1
g965.t1
MSTRG.30183.3
LOCMIG132150.1
XP_049832396.1
g5869.t1
Gsib_chr2_005186.1
Gsib_scaffold_75_000003.1
g10412.t1
Gsib_chr5_000902.1
XP_049837939.1
LOCMIG035760.1
LOCMIG035770.1
XP_049831452.1
g642.t1
LOCMIG133780.1
g6937.t1
LOCMIG039060.1
XP_049857354.1
Gsib_chr2_004568.1
XP_049857351.1
g6947.t1
XP_049857342.1
LOCMIG039170.1
Gsib_chr2_004566.1
LOCMIG039080.1
XP_049855197.1
g6941.t1
LOCMIG039110.2
XP_049857348.1
g6939.t2
LOCMIG039090.1
XP_049855604.1
g6943.t1
XP_049857346.1
LOCMIG039120.1
g6944.t1
XP_049857345.1
g6945.t1
XP_049857344.1
g6938.t1
LOCMIG039070.1
XP_049857350.1
g6951.t1
LOCMIG039200.1
XP_049857338.1
g6952.t1
XP_049857336.1
XP_049857337.1
g6956.t1
XP_049857330.1
g6953.t1
LOCMIG039220.1
XP_049857335.1
g6954.t2
LOCMIG039230.1
g6955.t1
XP_049857332.1
XP_049857333.1
XP_049857334.1
g7294.t1
XP_049851985.1
Gsib_chr2_001970.1
LOCMIG046690.1
XP_049851972.1
g7047.t1
XP_049851029.1
LOCMIG045120.1
g10344.t1
LOCMIG176540.1
XP_049840538.1
g10345.t1
LOCMIG176530.1
XP_049840542.1
g9927.t1
LOCMIG169670.1
XP_049838798.1
g10002.t1
LOCMIG176360.1
XP_049839609.1
LOCMIG176370.1
LOCMIG176380.1
XP_049839611.1
g9992.t1
LOCMIG176310.1
XP_049840833.1
g9993.t1
LOCMIG176320.1
g9995.t1
LOCMIG176330.1
XP_049839820.1
XP_049839819.1
g9998.t1
g10001.t1
LOCMIG176350.1
XP_049839718.1
g10000.t1
LOCMIG176340.2
g9955.t1
LOCMIG176030.1
XP_049838530.1
g7288.t1
LOCMIG046640.1
XP_04981988.1
g7289.t1
LOCMIG046650.1
XP_049851987.1
g7293.t1
LOCMIG046660.1
XP_049851986.1
0.2
